# Culturally appropriate organization of water and sewerage projects built through public private partnerships

**DOI:** 10.1371/journal.pone.0188905

**Published:** 2017-12-04

**Authors:** Jessica A. Kaminsky

**Affiliations:** Department of Civil and Environmental Engineering, University of Washington, Seattle, Washington, United States of America; Institut Català de Paleoecologia Humana i Evolució Social (IPHES), SPAIN

## Abstract

This paper contributes to the pursuit of socially sustainable water and sanitation infrastructure for all people by discovering statistically robust relationships between Hofstede’s dimensions of cross-cultural comparison and the choice of contract award types, project type, and primary revenue sources. This analysis, which represents 973 projects distributed across 24 low- and middle-income nations, uses a World Bank dataset describing high capital cost water and sewerage projects funded through private investment. The results show that cultural dimensions explain variation in the choice of contract award types, project type, and primary revenue sources. These results provide empirical evidence that strategies for water and sewerage project organization are not culturally neutral. The data show, for example, that highly individualistic contexts are more likely to select competitive contract award types and to depend on user fees to provide the primary project revenue stream post-construction. By selecting more locally appropriate ways to organize projects, project stakeholders will be better able to pursue the construction of socially sustainable water and sewerage infrastructure.

## Introduction

In the construction engineering and management literature, it is well established that the methods used to deliver infrastructure projects have implications for what is actually built and how it performs over time [1,2]. In this paper, I use this literature to seek culturally appropriate ways of organizing the construction of water and sewerage infrastructure around the world. In doing so, I follow a significant body of literature that describes the importance of considering cross-cultural differences in global undertakings. For example, Hall and Soskice [3] emphasize cross-national differences in economic and political institutions and their influence on policies and economic performance. Other researchers have developed frameworks for cross-cultural managerial challenges [4], workplace behavior [5], and leadership and organizational culture [6]. This body of literature has established the importance of considering cross-cultural differences in global undertakings. However, it has rarely been used to improve the process and outcomes of global infrastructure projects. As such, and given the increasingly global nature of the construction and engineering practice [7], I blend the literatures on project organization and cross-cultural comparison. The result is a contribution to theory that can address this practically important gap for public private partnerships in global infrastructure projects.

Since about the time of World War II, U.S. infrastructure construction projects have typically been delivered using what is called a design-bid-build (DBB) project delivery method. In this model, separate entities perform design and construction services, and the project is publically financed through the government [8]. More recently, however, public-private-partnerships (PPP) have become an increasingly common project delivery method [9]. PPP delivery methods are intended, among other things, to incentivize private investment in public infrastructure. The firm involved in the design and construction helps finance the project, profiting later by taking some form of ownership or operational responsibility for the constructed asset. The private entity is therefore financially motivated by both the infrastructure’s creation and its performance over time. Due to this financial incentive for long-term performance, this approach has been characterized as seeing infrastructure as a long-term service rather than as a one-off product [10].

The use of PPP in infrastructure construction projects is reflective of recent trends towards private, market-based solutions to the provision of public infrastructures [11–13] such as the water and sewerage projects that interest us here. As such, this project delivery typology from the construction engineering community complements the well-established participatory development [14] discourse common in international development. Participatory development seeks to improve the chances of building socially sustainable infrastructure, used here in a limited sense to mean infrastructure that is used and maintained over time by host communities. As it is enacted, this approach requires the use of private resources from local businesses and residents to support the initial construction and ongoing provision of economically sustainable infrastructure services. Thus, both PPP project delivery methods and participatory development methods use private investment as a means to build new infrastructure and to incentivize its performance over time [15,16]. A common although not absolute difference between these two is that participatory development schemes have tended to privilege the small and local [17] for both solutions and finance, while PPP methods have tended to privilege larger projects with external advisors and finance. This is an important difference given that there is a known liability of foreignness [18] in global projects. Still, both PPP and participatory development strategies seek to move away from a model of direct government finance of infrastructure.

Critics of participatory development methods are concerned about the effects of shifting investment costs to the poor [19], and they question if participation is used to minimize dissent or to enhance accountability of the powerful to the poor [20]. Similarly, serious concerns remain regarding how the private profit incentives inherent to PPP may or may not dissuade the provision of basic infrastructure services to the poor [21,22]. But beyond these philosophical debates, the evidence also remains mixed on the effects of both participation and private investment in infrastructure construction and performance [23–26]. For example, while noting the potential of private investment in public infrastructure projects, the World Bank reports issues of conflicts, high incidence of renegotiation, and abandonment of infrastructure by the private partner [27]. Given the increasing attention to PPP in both domestic and international infrastructure construction [28], the research community has expended considerable effort studying them. Researchers are exploring, for example, the (still contested) claims that using private resources and alternative project delivery structures creates infrastructure that otherwise would not be built, or that by changing the incentive structure of the constructing firm we can improve what is built [8,11]. While these claims have yet to be definitively settled, there is growing empirical and intuitively appealing evidence that the context in which PPP are built strongly influence their chances of success or failure [9,15,28].

Building on this past work, and using World Bank terminology [29], in this paper I ask if national level cultural trends explain variation in:

- (Research Question 1) how international water and sewerage construction projects with private investment are built (different *project types*),
- (Research Question 2) the ways in which private involvement is contracted (direct vs. competitive *contract award types*), and
- (Research Question 3) as private investment requires profitability, the ways in which *revenue streams* are generated by the infrastructure post-construction.

As I will show, this analysis uncovers statistical evidence of the previously theorized importance of cultural context to water and sewerage construction project organization. In practical terms, the statistical relationships discovered here mean that various aspects of water and sewerage project organization are more or less culturally appropriate, and thus are more or less socially sustainable, in different national contexts. I answer the three research questions using an international dataset of 973 high capital cost water and sewerage projects that leveraged private investment. I use Hofstede’s [5] cultural dimensions to operationalize cross-national cultural differences; these are discussed below. Next, I discuss the particular aspects of project organization addressed in the research questions. This is followed by a description of the data underpinning the multinomial logistic regression analysis used here. Finally, I present the statistical results and discuss what they mean for the practice and theory of global infrastructure project organization.

### Hofstede’s dimensions

Culture—an admittedly uncomfortable construct [30]—is recognized as important to water and sanitation infrastructure [31,32]. However, it is not an easy construct to measure, particularly for the purpose of cross-cultural comparison. One solution to this difficulty in the measurement of social difference is through the use of quantitative dimensions that enable comparison through simplifications. For example, Markus and Hamedani [33] note that “cultural differences may reflect underlying basic value orientations, beliefs, and worldviews prevalent in a context; however, these differences can be best and most parsimoniously captured by identifying and describing cultures according to where they fall along a series of dimensions”.

The relative ease of quantitative dimensional analysis means that existing dimensional frameworks have been widely used both in practice and theory. In the Harvard Business Review online, for example, there are currently almost 80 references to Hofstede’s dimensional framework [5,34]. Still, there are certainly competing sets of quantitative cultural dimensions [6,35], and any number of more localized qualitative approaches have been used in construction engineering research [36]. By selecting Hofstede’s framework from these possibilities, I do not intend to imply that these dimensions are the only possible way to think about culture and water and sanitation projects. I claim only that the framework’s wide previous use gives it strong construct validity that better enables the interpretation and practical application of results. Future research that uses different approaches to cross-cultural comparison is needed to both problematize and complement the results presented here.

Hofstede’s cultural dimensions [5] are used by both academics and the global project practice [37] to compare nations based on aggregate preferences. In the early 1970s, Hofstede gained access to over 80,000 survey responses from IBM employees. He used these surveys to create a set of four dimensions that he claimed describe fundamental cultural preferences. Importantly, the numeric scores do not imply better or worse, but rather attempt to measure the scale of difference. These four dimensions are [5]:

- *Individualism vs*. *Collectivism* (IDV): “a preference for a loosely-knit social framework in which individuals are expected to take care of only themselves and their immediate families” vs. a “preference for a tightly-knit framework in society in which individuals can expect their relatives or members of a particular in-group to look after them in exchange for unquestioning loyalty.”
- *Masculinity vs*. *Femininity* (MAS): a “preference in society for achievement, heroism, assertiveness, and material rewards for success. Society at large is more competitive.” vs. a “preference for cooperation, modesty, caring for the weak and quality of life. Society at large is more consensus-oriented.”
- *Power Distance Index* (PDI): “the degree to which the less powerful members of a society accept and expect that power is distributed unequally. The fundamental issue here is how a society handles inequalities among people.”
- *Uncertainty Avoidance Index* (UAI): “expresses the degree to which the members of a society feel uncomfortable with uncertainty and ambiguity.”

In academic circles, the dimensions themselves have been a source of significant debate, with critiques including assertions of the problems inherent to generalizing to the nation state, the limitations of assuming IBM employees to be representative of national populations, or the improbability of being able to represent the complexities of culture with numbers [37]. Still, researchers have generally found Hofstede’s dimensions to be a useful, though certainly imperfect, model for understanding cross-cultural difference [38,39]. In addition, recent research has been reasonably successful in validating Hofstede’s dimensions against more recent and more comprehensive surveys [40,41]. As such, Hofstede’s dimensions are counted as classic social theory, with wide applications in diverse fields such as management studies, environmental studies, and construction engineering [42–46].

### Project organization

Three key organizational strategies used in infrastructure construction—each defined below—are examined here. These three are the contract award method, the primary revenue source, and the project type. Chosen based on data availability, these three organizational strategies are certainly not the only ways to characterize PPP structure [47]. They are, however, particularly fundamental features of PPP, as evidenced by their selection for the World Bank PPI database [48] used in this analysis.

### Contract award method

As defined by the World Bank [29], projects using *competitive bidding* invite bids from competing private organizations through an open advertisement that describes the project scope, contract terms and conditions, and how the received bids will be evaluated. In a contract, projects that use *direct negotiations* award a contract to a private organization, and forgo the competitive bidding process [49]. The literature suggests that contracts should be understood as a management tool used to structure relationships [50]. As such, research has indicated that contract award methods should be matched with project types or owner requirements [51]; some researchers have attempted to link contract structure to construction project performance [52]. In this body of knowledge, competitive bids have been linked to understanding construction as a commodity that should be purchased at the lowest possible price, while direct negotiations structure construction as a service and a relationship [51].

### Primary revenue source

Private entities invest in infrastructure in order to make a profit. As such, these projects must necessarily have a method of generating revenue. In the dataset analyzed here [49], the primary revenue streams were structured as purchase agreements, annuity/available payments, user fees, and an unspecified other. *Water purchase agreements* mean that the host government agrees to purchase water from a private provider for a set period of time. This guarantees a revenue stream for the private operator, and means that the government purchases this service rather than the water infrastructure itself. In contrast, *annuity/available payment* structures mean that a government agrees to make a predefined, periodic payment to a private firm in exchange for the provision of infrastructure services. In yet another revenue model, a third type of projects generate revenue by depending exclusively or mainly on *user fees*, or on payments from individuals on a pay-per-use basis.

### Project type

Project delivery methods describe how projects are designed and constructed As defined by the World Bank [29], in *Greenfield Projects* private entities are involved in both building and operating infrastructure. In this dataset, greenfield projects include two project types—build, operate, and transfer (BOT); and build, own, and operate (BOO). BOT projects involve “a private sponsor [that] builds a new facility at its own risk, owns and operates the facility at its own risk, then transfers the facility to the government at the end of the contract period” [29]. In contrast, BOO projects involve “a private sponsor [that] builds a new facility at its own risk, then owns and operates the facility at its own risk” [29].

The other project types represent private investment in infrastructure that does not include the construction of new facilities. For example, in *Divestiture Projects* a private entity buys a stake in state-owned infrastructure. Such purchases can be full or partial divestitures. In full divestiture, the government transfers 100% of equity to a private entity; in partial divestiture, only part of the equity is transferred, with the state retaining partial ownership. In addition, the World Bank tracks *Brownfield Projects*, where private investors invest funds to expand or rehabilitate a facility, recoups the investment through operation of that facility, and then transfers it back to the government at the end of the contract period. In this dataset, brownfield projects include the following types: rehabilitate, operate, and transfer (ROT), wherein “a private sponsor rehabilitates an existing facility, then operates and maintains the facility at its own risk for the contract period” [29]; rehabilitate, lease or rent, and transfer (RLT), wherein “a private sponsor rehabilitates an existing facility at its own risk, leases or rents the facility from the government owner, then operates and maintains the facility at its own risk for the contract period” [29]; and build, rehabilitate, operate, and transfer (BROT), wherein “a private developer builds an add-on to an existing facility or completes a partially built facility and rehabilitates existing assets, then operates and maintains the facility at its own risk for the contract period” [29]. The final project type represented in this dataset is *Management and Lease Contracts*. In these projects the state retains ownership of infrastructure assets, but a private entity is responsible for the management of the asset [29].

## Methods

### The data

Updated every six months, the World Bank’s Private Participation in Infrastructure (PPI) database has data on over 7,000 infrastructure projects in 139 low- and middle-income nations [48]. These are high capital cost, public infrastructure projects that have at least 20% private participation in any sphere of ownership, finance, or operation. PPP projects are a subset of the projects represented in this database [53]. If they are operating outside of their home country, state-owned enterprises are considered to be private companies. Some projects are entirely private, while others have both public and private involvement. As of the date of this analysis, the database includes project from 1983 to 2015. In the PPI database, investments with separate financing packages, even if invested in the same facility, are counted as separate projects [54]. From this dataset, I extracted all water and sewerage projects. These are defined as potable water generation and distribution, and sewerage collection and treatment.

Hofstede’s dimensions [55] were filtered to match national scores with nations included in the PPI dataset. This combination resulted in a dataset describing 973 projects from the following 24 nations: Argentina, Brazil, Bulgaria, Chile, China, Columbia, Ecuador, Guatemala, India, Indonesia, Malaysia, Mexico, Morocco, Panama, Peru, Philippines, Romania, Russia, Serbia, Thailand, Turkey, Uruguay, Venezuela, and Vietnam. Many of these nations used different contract award methods, revenue sources, and project types in different projects. As a control variable in the analysis, I include 2013 gross domestic product (GDP) data from the World Bank [56]. A key limitation of this research is that the PPI database of projects is a compilation of publically available data, meaning there is risk of inaccuracies [54]. A second and related limitation is that this database poorly represents small projects, as there is less public information reported on these projects.

### Data analysis

To analyze this data, I used multinomial logistic regression (also called a discrete choice model) using STATA 14.2 *mlogit* commands. This analysis approach enables me to determine if each response variable (for example, the choice of bid criteria) is influenced by the set of predictor variables (in this analysis, the cultural dimensions and GDP). Three separate analyses were run to determine if the cultural dimensions were statistically significant predictors of the contract award method, the primary revenue source, and the project type. The results are presented separately below.

## Results

In this section, I present statistically significant relationships between Hofstede’s cultural dimensions and the three organizational choices in PPP water and sewerage projects (contract award method, primary revenue source, and project type). After detailing these statistical findings, I summarize and discuss these results, noting that future research is needed to confirm the proposed explanations of the observed statistical relationships.

### Contract award method

The contract award method is a record of how project contracts are awarded. At a high level, these may be divided into competitively awarded contracts, and those that were instead negotiated with a pre-selected set of firms. For this analysis, I omitted the projects with no data on the contract award method (resulting in a dataset of 599 projects) and set Direct Negotiation as the base case. In other words, any statistical significance reported below indicates that a particular cultural dimension is a statistically significant predictor for the choice between Direct Negotiation and the other (competitive) contract award methods in the dataset. For water and sewerage projects in the PPI dataset, there are two competitive award types and one direct award type. The competitive award types are presented only in aggregated form due to a very small number of water and sewerage projects in the competitive negotiation category. Therefore, the results may be understood as indicating how aggregate cultural preferences influence the choice of competitive vs. negotiated contract award methods.

As shown in Table 1, PDI, IDV, and MAS are statistically significant predictors of whether a competitive or direct contract award method will be used, after controlling for GDP. Competitive bidding is statistically associated with low PDI, high IDV, and high MAS. In other words, nations with cultural preferences for less hierarchy, individualism, and competitive relationships are more likely to use competitive bids. In contrast, preferences for hierarchy, collectivism, and collaborative relationships tend to lead to direct contract award methods. It is worth reiterating that these results do not mean that contexts with these cultural descriptors use only the predicted contract award method, but rather that this is what they use more often.

**Table 1:** Results of Multinomial Logistic Regression, Contract Award Method on Water and Sewerage Projects.

Hofstede’s Dimension	Competitive Bidding or Competitive Negotiation (599 projects)
PDI	-0.19*** / (0.04)
IDV	0.16** / (0.05)
MAS	0.15*** / (0.04)
UAI	0.04 / (0.02)
GDP	-0.00* / (0.00)
Intercept	3.70 / (2.84)

Total n = 599 projects.

Notes: Reference category for equation is Direct Negotiation (122 projects).

rrr^p-value^ / (standard error).

*p<0.05.

**p<0.01.

***p<0.001.

### Primary revenue source

The primary revenue source variable captures the way in which private profit is created in an infrastructure project. For this analysis, I omitted the projects with no data on the primary revenue source, resulting in a dataset of 973 projects. Of these projects, 439 were classified as *other*, meaning they did not fit into the reported categories. These 439 projects were aggregated along with the annuity and purchase agreement categories to form a category of all projects that do not depend on user fees as the primary source of income. For this analysis, the base case is User Fees as the source of primary revenue, and any statistically significant results discussed here mean that there are cultural drivers differentiating between end-use customer fees and other models. This choice was made because of the ongoing debates in the literature regarding the challenges of the scale and collection of user fees to support water and sewerage infrastructure. For example, Rayner et. al. [57] report users in Haiti object to paying for water, and that they fear misuse of fees, while Hopkins [58] seeks to optimize user willingness to pay against the ubiquity of handpump availability.

As shown in Table 2, high MAS scores (preferences for competitive relationships) predict the choice of annuities instead of user fees, while low IDV and low UAI scores (preferences for collectivism and uncertainty embracing) predict the choice of purchase agreements instead of user fees. The results from the aggregated category show that preferences for collectivism and competitive relationships (low IDV and high MAS) drive the use of user fees over other revenue models. While UAI does not show a statistically significant relationship, the p value was very close to the cutoff at 0.07. If we relax the significance standards this far, nations that prefer to avoid uncertainty tend to generate revenue via user fees. Surprisingly, this suggests that user fees are perceived as less risky than other methods of revenue generation. This may be because governments dictate the form of public-private relationships, and are not responsible for payments in user fee revenue models, thus shifting risk to the private entity. Future research is needed to explore this surprising relationship.

**Table 2:** Results of Multinomial Logistic Regression, Primary Revenue Source from Water and Sewerage Projects.

Hofstede’s Dimension	Annuity / Available Payment (74 Projects)	Purchase Agreements (298 Projects)	All, Not User Fees (973 Projects)
PDI	0.05 / (0.03)	0.02 / (0.03)	0.01 / (0.01)
IDV	-0.03 / (0.02)	-0.09*** / (0.02)	-0.07*** / (0.01)
MAS	0.10*** / (0.02)	0.04 / (0.02)	0.04*** / (0.01)
UAI	-0.02 / (0.02)	-0.05** / (0.01)	-0.02 / (0.01)
GDP	-0.00 / (0.00)	-0.00 / (0.00)	-0.00* / (0.00)
Intercept	-8.18* / (3.21)	2.01 / 2.43	1.87 / (1.19)

Total n = 534 projects (excluding ‘other’ types) or 973 Projects (including ‘other’ types).

Notes: Reference category for equation is User Fees (162 projects).

rrr^p-value^ / (standard error).

*p<0.05.

**p<0.01.

***p<0.001.

### Project type

For this analysis, all projects in the dataset had data on the project type, resulting in the analysis of 973 projects. The base case was set as a *Greenfield Project*, which here means that private entities were involved in both building and operating infrastructure. The other project types represent private investment in infrastructure, excluding the construction of new facilities. In *Divestiture Projects*, for example, a private entity buys a stake in state-owned infrastructure. In *Brownfield Projects* private investors invest funds to expand or rehabilitate a facility, recoup the investment through operation of that facility, and then at the end of the contract period transfer it back to the government. Finally, in *Management and Lease Contracts* the state retains ownership of infrastructure assets, but a private entity is responsible for the management of the asset.

As shown in Table 3, pursuing PPP brownfield or divestiture projects instead of greenfield projects is predicted by low PDI and low MAS scores. In addition, IDV and UAI scores drive projects towards either brownfields (low IDV and low UAI, or individualism and uncertainty embracing) or management and lease contracts (high IDV and high UAI, or collectivism and uncertainty embracing). Interestingly, higher GDP predicts the choice of brownfield rather than greenfield projects. This may be due to the greater existence of potential brownfield assets in wealthier contexts.

**Table 3:** Results of Multinomial Logistic Regression, Project Type of Water and Sewerage Projects.

Hofstede’s Dimension	Brownfield Project (431 Projects)	Divestiture (53 Projects)	Management and Lease Contract (105 Projects)	All, Not Greenfield (589 Projects)
PDI	-0.04*** / (0.01)	-0.09** / (0.03)	0.03 / (0.02)	-0.03** / (0.01)
IDV	0.02* / (0.01)	0.06 / (0.06)	-0.06** / (0.02)	0.01 / (0.01)
MAS	-0.04*** / (0.01)	-0.24*** / (0.07)	-0.02 / (0.01)	-0.04*** / (0.01)
UAI	-0.02* / (0.01)	-0.02 / (0.03)	0.0** / (0.01)	-0.01 / (0.01)
GDP	0.00*** / (0.00)	0.00* / (0.00)	-0.00 / (0.00)	-0.00** / (0.00)
Intercept	7.20*** / (1.35)	14.55** / (4.21)	-3.00 / (1.84)	6.23*** / (1.24)

Total n = 973 projects.

Notes: Reference category for equation is Greenfield Project (384 projects).

rrr^p-value^ / (standard error).

*p<0.05.

**p<0.01.

***p<0.001.

## Discussion

Table 4 summarizes the statistically significant relationships detailed above. In this discussion, I use two approaches to think about the observed relationships. One is to use these results as direct guidance to select more culturally appropriate structures for water and sewerage projects. For example, a private entity, donor agency, or multilateral looking for investment opportunities in a particular country could look for that nation’s Hofstede scores [55] to help understand if new construction (greenfield projects) or rehabilitation of existing assets are more likely to be locally acceptable. For example, this would suggest that, all else being equal, nations with high scores in PDI (e.g. Malaysia or Guatemala) would be more likely to prefer private investment in greenfield projects than would nations with low PDI scores (e.g. Lithuania or Costa Rica). This sort of application of Hofstede’s framework is common and often productive in both the global projects literature and business practice [37]. Still, it must be done with care. Culture is certainly not the only factor impacting the structure of infrastructure projects. As evidence of this, we may review the statistically significant relationships between national GDP and contract award methods, primary revenue source, and project type. In addition, it is deeply problematic to apply national level trends such as those presented here to any individual instance. Theoretically, this is known as the ecological fallacy—aggregate trends cannot be assumed to predict the behavior of any particular individual [59]. Given these and other limitations, this type of application should be done as a screening level tool only, in combination with other types of evidence that can more accurately localize the global trends reported here.

**Table 4:** Statistically significant results summary.

Hofstede’s Dimension	Contract Award Method	Primary Revenue Source	Project Type
**High PDI (hierarchy)**	Direct Negotiation	-	Greenfield
**High IDV (individualism)**	Competitive Bidding	User Fees	-
**High MAS (competition)**	Competitive Bidding	Not User Fees	Greenfield
**High UAI (uncertainty avoiding)**	-	-	-
**High GDP**	Direct Negotiation	User Fees	Not Greenfield

A second and less problematic way to interpret these results is to use them to better understand the cultural work performed by these various ways to structure infrastructure projects. These insights may then be more directly applied to improve individual project outcomes by providing a rationale for the project organization based on project needs and stakeholders. For example, while much of the data on contracting is considered proprietary and is therefore unavailable to researchers for analysis, competitively awarded contracts are generally thought to result in the selection of the low-cost bidder and to avoid favoritism, while direct negotiation contracts are generally thought to be faster and to produce higher quality outcomes. As seen in Table 4, competitive contract award methods are more often used in contexts that prefer low hierarchy/privilege for the elite, individualism, and competition rather than collaboration (low PDI, high IDV, and high MAS). The results presented here provide empirical evidence that, as we might have hypothesized, competitive contracts are used to level the playing field for less powerful or less established entities. In these contexts, it is believed that competition leads to better results by avoiding favoritism and choosing the best available entity to deliver a project. In contrast, direct negotiation is used in contexts that believe the elite deserve privileges. In these contexts, the underlying understanding is that particular entities are elite because they deliver better results. It is important to emphasize that neither of these organizational approaches, nor any other described in this paper, are wrong or right. Any of them are capable of delivering excellent infrastructure projects. These approaches to project organization all seek to achieve the best project outcomes. However, local understandings differ regarding how to do this, or even of how to measure what we mean by a best project outcome. In the development discourse, we might say that these approaches reflect different theories of change.

We might expect that the selection of projects’ primary revenue sources would be the pure result of a negotiation between private partners seeking to maximize economic returns and the public sector goals of low costs. Instead, the data presented here show that individualistic contexts (high IDV) and those that tend towards collaborative relationships (low MAS) prefer to depend on revenue from end-user fees. Contexts that prefer collectivism (low IDV) and tend to embrace competition and conflict (high MAS) prefer to avoid end-user fees in favor of other revenue sources. This suggests that user fees do the cultural work of enabling individual households to collaborate to achieve water or sewerage services (high IDV, low MAS). Other, non end-user fee approaches to providing revenue for this critical infrastructure are understood as more broadly collective (low IDV). For example, such approaches may provide service to the poorest of the poor (those who would have trouble making individual payments) while also providing a structure for managing culturally acceptable conflict (high MAS) regarding payment of those fees. The water and sanitation in international development communities are often based on the assumption that collective contexts are more likely to prefer community-managed systems with individual tariff schemes; our data problematizes that discourse. A caveat to this critique is that the data used here does not represent small projects well, due to the limited media coverage of such projects. In addition, the data do not include projects that are entirely government funded. Despite these limitations, however, the data provide some evidence regarding contexts in which user fees are—and are not—culturally appropriate ways to gather water and sewerage project revenue.

For selecting project types, more competitive (high MAS) and hierarchical (high PDI) settings are both more likely to use private funds to build greenfield projects. As described above, these are projects that construct and then operate new infrastructure, as opposed to rehabilitating existing infrastructure assets. High MAS scores describe contexts where competition and external achievement are preferred to collaboration [5]. As such, it is possible that in these contexts, private entities are drawn to creation and control through new construction, rather than participating in repairs and operations of what was created by someone else. The PDI dimension describes how nations understand hierarchical inequalities [5]; the data show that contexts that are more comfortable with inequalities (high PDI) tend to use private funds to build greenfield projects. While our dataset cannot confirm this proposed explanation of the observed relationships, as PPP projects are expected to generate profits for the private investors, they would reasonably be expected to target relatively wealthier customers. For example, this might mean building a sewer to a rich neighborhood and bypassing a poor one. In low PDI contexts, where this type of hierarchy is seen as less acceptable, private investment tends to flow instead to improving and operating existing infrastructure rather than creating new assets that private finance might reasonably intend for the wealthy who are better able to pay for the new infrastructure services. Table 3 further complicates these project type summary relationships. For example, the data show that uncertainty accepting contexts (low UAI scores) prefer to use private funds in brownfield projects, while uncertainty avoiding contexts (high UAI scores) prefer to use private funds in management and lease projects. This suggests that the government, the private investor, or both, perceive it to be less risky for the government to retain ownership of the infrastructure assets. In another example, in individualistic contexts with high IDV scores there is a preference to use private funds in brownfield projects, while in collectivistic contexts with low IDV scores, there is a preference to use private funds in management and lease projects. Here the data suggest that in individualistic contexts, private investors prefer to have individual control over the asset through an ownership stake, while in more collectivistic contexts the private investors appear to be more comfortable with more substantial government involvement.

## Conclusion

The data show that Hofstede’s cultural dimensions explain variation in the selection of contract award methods, primary revenue source, and project type for large water and sewerage infrastructure projects in 24 low- and middle-income nations around the globe. I understand these results to be evidence that different ways of organizing water and sewerage projects are not culturally neutral; instead, these methods are more or less culturally appropriate depending on the context of the project. It should be reiterated that all the ways of organizing projects analyzed here are capable of delivering quality infrastructure, and that in most contexts a variety of strategies are used. The findings of this research do not imply that certain contexts yield a preference for better or worse organizational strategies. Rather the findings describe preferences regarding different, equally viable, and equally (if differently) rational ways of undertaking water and sewerage infrastructure projects. Still, the results presented here should not be interpreted as evidence regarding the potential of the various organizational strategies for procedural corruption or of their relative potential for good project outcomes. These issues must be left for future research, which should aim to provide this practically important knowledge to people seeking to build socially sustainable global infrastructure projects. In addition, while it is analytically useful to separate cultural constructs, in practice different aspects of culture overlap and influence each other. As such, a valuable undertaking would be research that employs a set theoretic methods [60,61] that looks for combinations of the cultural dimensions and their influence on PPP. Additionally, as the data show that culture matters for PPP structure, we would expect that multi-cultural teams would also influence projects. Future work should explore this point, which links to the literature on team diversity, foreignness, and project outcomes [18,62]. More broadly, given the statistically significant results presented here future research should explore if and how cultural dimensions may be relevant to other aspects of WASH project organization such as governance structures or the success rates of approaches such as community led total sanitation.

The results presented above, in combination with Hofstede scores for particular nations, may serve as an early screening tool for how culturally appropriate a particular project organization method is for a particular water or sewerage project. Global businesses have productively used Hofstede’s scores in this manner for decades. However, it must be emphasized that culture is not the only contributing factor to these choices, nor does a national descriptor of culture accurately describe all people within that nation. Cultural identities are importantly multiple, diverse, and dynamic. As such, the strongest contribution of this work is its discovery of the cultural work performed by the use of particular contract award methods, primary revenue sources, and project types. For example, the data show that water and sewerage projects with private investment generate revenue through user fees in individualistic contexts, and avoid the use of user fees in more collective contexts where (for example) it is less culturally acceptable to risk individuals being unable to afford individual payments. In another example, the data suggest that selecting user fees instead of other revenue structures is done to shift project risk from public to private sectors. Future qualitative research is needed to confirm these and other proposed explanations of the observed statistical relationships. Nonetheless, the data presented here suggest relationships between deep-seated cultural preferences and ways of organizing private investment in water and sewerage projects. These relationships should be considered in the pursuit of socially sustainable water and sewerage infrastructure. By framing and structuring WASH projects in locally and culturally meaningful ways, we may be able to add to its perceived legitimacy [63] and thereby increase both its uptake (or, diffusion [64]) and longevity of use (or, institutionalization [65]).
